# Graphene Functionalized Scaffolds Reduce the Inflammatory Response and Supports Endogenous Neuroblast Migration when Implanted in the Adult Brain

**DOI:** 10.1371/journal.pone.0151589

**Published:** 2016-03-15

**Authors:** Kun Zhou, Sepideh Motamed, George A. Thouas, Claude C. Bernard, Dan Li, Helena C. Parkington, Harold A. Coleman, David I. Finkelstein, John S. Forsythe

**Affiliations:** 1 Department of Materials Science and Engineering, Monash Institute of Medical Engineering, Monash University, Melbourne, VIC, Australia; 2 Australian Regenerative Medicine Institute, Monash University, Melbourne, VIC, Australia; 3 Department of Physiology, Monash University, Melbourne, VIC, Australia; 4 The Ritchie Centre, Hudson Institute of Medical Research, Monash University, Melbourne, VIC, Australia; 5 The Florey Institute of Neuroscience and Mental Health, The University of Melbourne, Melbourne, VIC, Australia; Michigan Technological University, UNITED STATES

## Abstract

Electroactive materials have been investigated as next-generation neuronal tissue engineering scaffolds to enhance neuronal regeneration and functional recovery after brain injury. Graphene, an emerging neuronal scaffold material with charge transfer properties, has shown promising results for neuronal cell survival and differentiation *in vitro*. In this *in vivo* work, electrospun microfiber scaffolds coated with self-assembled colloidal graphene, were implanted into the striatum or into the subventricular zone of adult rats. Microglia and astrocyte activation levels were suppressed with graphene functionalization. In addition, self-assembled graphene implants prevented glial scarring in the brain 7 weeks following implantation. Astrocyte guidance within the scaffold and redirection of neuroblasts from the subventricular zone along the implants was also demonstrated. These findings provide new functional evidence for the potential use of graphene scaffolds as a therapeutic platform to support central nervous system regeneration.

## Introduction

Neuronal regeneration and functional recovery after brain injury and degenerative disease are limited due to inhibitory molecules at the lesion site [1,2], lack of trophic support, in the case of significant tissue loss [3], and the post-mitotic stage of adult neurons [4]. To promote neuronal regeneration under inhibitory conditions, tissue engineering scaffolds need to provide a supportive micro-environment to encourage endogenous nerve migration, while controlling inflammatory cell infiltration. Many recent engineered scaffolds have been manufactured as physical forms of nano- or micro-fibers and hydrogels [5–9], with the intent to mimic the natural extracellular matrix microstructure [8,10,11]. Numerous biomolecules have been incorporated into these scaffolds, either as precursors during scaffold synthesis [12,13] or post-treatment, such as surface immobilization [14,15]. The incorporated biomolecules have been employed to regulate neuronal survival, cellular behaviour and inflammatory response suppression [6,7]. We have previously demonstrated that biomaterial scaffolds can lower the inflammatory response and facilitate the regeneration [5,7] with a well-tuned microstructure and the controlled release of neurotrophic factors.

Recently, scaffolds for neuronal tissue engineering have been rendered electroactive, using materials such as poly-(3,4-ethylenedioxythiophene) (PEDOT) and graphene. This treatment supports electrical interactions within neuronal cell networks, and improves cell guidance [16–22]. Graphene-based materials are already widely used in biomedical applications, such as cell and tissue imaging [23–25], cancer therapy [26–28] and neuronal tissue regeneration [17,29,30]. While some studies have shown that soluble forms of graphene nanosheets can be cytotoxic to specific cell types [31–33], other studies have reported potential benefits of graphene when used with scaffolds and substrates for neuronal tissue *in vitro*, such as promotion of neuronal cell survival, proliferation, network signalling and differentiation [18,34–36]. Several mechanisms have been proposed to explain these bioactive properties of graphene materials, including direct electrical coupling between the graphene and cells [18], or molecular interactions mediated by functionalized proteins at the graphene-cell interface [34]. Previous reports have thus explored surface deposition of graphene onto 2D or 3D scaffolds by chemical vapor deposition (CVD) [17,30], however, these typically require template etching or film transfer using expensive and high temperature processing techniques. In contrast, we previously developed a coating strategy using colloidal graphene incorporated by layer-by-layer (LbL) assembly onto electrospun poly-ε-caprolactone (PCL), which successfully renders neuronal scaffolds bio-functional and electroactive [37]. This approach is suitable for low melting-temperature polymeric scaffolds with complex microstructures, over a large scale. While such scaffolds are beneficial to neuronal function *in vitro*, their *in vivo* performance has not been very well characterized, leaving a large gap in the knowledge of their compatibly with the brain and thus putative suitability as a therapeutic option.

Herein, we report the use of graphene polyelectrolyte multilayer formation as an electro-active substrate on electrospun PCL microfiber scaffolds for brain repair. Graphene functionalized scaffolds were implanted into the striatum of adult rats, to assess the inflammatory responses of microglia and astrocytes. The time frame and morphology change of these inflammatory cells were also monitored. In addition, graphene functionalised scaffolds were implanted to intercept the subventricular zone (SVZ) to evaluate whether they could encourage guidance of resident neuroblast cells in adult neurogenesis.

## Materials and Methods

### gPEM-PCL scaffolds preparation

Detailed electrospinning processing and graphene-polyelectrolyte multilayer (gPEM) build-up have been described previously [37]. Graphene oxide preparation has been described elsewhere [38].

Briefly, purified graphene oxide was suspended in deionized water (2 mg/mL), followed by exfoliation using an ultrasonication probe (750W, Sonics & Materials Inc., US) at 50% amplitude for 30 min. 175 μL ammonia solution (28 w/v%, Ajax Finechem, Australia). 200 μL hydrazine solution (35 wt% in water, Sigma-Aldrich, NSW, Australia) was then added to 20 mL ultrasonicated graphene oxide allowing chemical reduction to proceed at 95°C in a water bath for 3 hr. Electrospun PCL (MW: 70,000–90,000, Sigma-Aldrich) micro-fibrous scaffolds were prepared as follows: 11 w/v% PCL was dissolved in 3:1 chloroform/methanol with 0.06 w/v% NaCl solution. Electrospinning was performed using a 2.2D-350 electrospinner (Yflow Nanotechnology Solutions, Spain) using a pumping rate of 2 mL/h, a working distance of 10 cm and voltage of 11 kV. A rotation speed of 800 rpm was used for the collection mandrel to achieve aligned microfiber morphology. Electrospun PCL was primed in 20 mg/mL polyethyleneimine (MW: 750,000, Sigma-Aldrich) in PBS (pH 7.4) for 2 hr, and was then alternately incubated in heparin (5 mg/mL, Sigma-Aldrich)/graphene (0.5 mg/mL) and poly-L-lysine (PLL, 1 mg/mL, Sigma-Aldrich) PBS solutions six times, with the outermost (terminating) layer being the sixth poly-L-lysine (gP6) or graphene/heparin (gH6); PBS rinsing was used as an intermediate step. Scaffolds prepared following the above protocol, but without addition of graphene mixed in the heparin solution, served as graphene-free controls (P6, H6). The resulting gPEM-PCL (gP6, gH6) test scaffolds, and PEM-PCL (P6, H6) control scaffolds were then crosslinked in 2 wt% 1-ethyl-3-(3-dimethylaminopropyl) carbodiimide/PBS solution at pH 7.4 (EDC, Sigma-Aldrich), followed by thorough PBS rinse.

### *In vivo* study

gPEM-PCL and PEM-PCL scaffolds were sterilized in 80% ethanol, followed by air drying for 30 min. Sterilized scaffolds were cut into 5×2 mm strips and then rolled manually into scroll-like structures 5 mm in length). Scrolled scaffolds were then loaded into 21-gauge needles, which were soaked in sterile PBS for 1 hr prior to implantation. 4 male Wistar rats (approximate weight: 300 g) were used per experimental group (all methods conformed to the National Health and Medical Research Council published Code of Practice for the use of animals in research, and were approved by the Howard Florey Institute Animal Ethics Committee). Each rat was given 15 mg/g atropine (per gram of animal weight, Pfizer Pty Ltd, NSW, Australia) and 1mg/g xylazine (Troy Laboratories Pty Ltd, NSW, Australia) in saline solution (Baxter, NSW, Australia), followed by anaesthetic using 3% isofluorane in oxygen at 1.0 L/min. Following anesthesia, the rat skull was fixed on a stereotactic frame. For striatum implants, two craniotomies were performed on both sides of the same skull (1.0 mm anterior of bregma; 2.5 mm laterally from the midline). For scaffold implantation, the loaded needle was vertically inserted 6 mm below the skull into the brain tissue and maintained in position by the plunger, while the needle was slowly retracted. For SVZ implants, one craniotomy was performed at 1.0 mm anterior of bregma, 3 mm lateral from the midline. The gP6 scaffold was then implanted at a 25° angle to the vertical in the coronal plane. Animals were allowed to recover for designated time points and then humanely killed by injection of 0.5 mg/g sodium pentobarbitone (Virbac, TX, USA). Each animal was immediately perfused with 250 mL of chilled PBS, followed by 4% chilled paraformaldehyde (PFA, Sigma-Aldrich, USA). Whole brains were then removed from dissected skulls, soaked in 4% PFA at 4°C overnight and transferred to 30% sucrose/PBS solution the following day, followed by a further 2 days of storage at 4°C. All frozen brains were sectioned transversely at 30 μm thickness using a cryostat (Leica, Germany). Sections were mounted onto microscope slides (Superfrost Plus, Germany) for immuno-staining.

### Immunohistochemistry

Brain sections were further fixed with 4% paraformaldehyde for 1 min, followed by a PBS rinse, and then blocked with 10% normal goat serum (NGS, Vector Laboratories Ltd. UK), 1% BSA (Sigma-Aldrich) and 0.2% Triton-X100 in PBS for 1 hr at room temperature. Mouse-anti-SMI 32 (1:750, SMI-32P, Covance, Australia), rabbit-anti-GFAP (1:1000, Z0334, Dako, Australia), rabbit-anti-Iba1 (1:250, 019–19741, Wako Pure Chemical Industries, Japan), rabbit-anti-DCX (1:500, 4604S, Cell Signaling, Australia) were all used as primary antibody markers for astrocytes, microglia and neuroblasts, respectively. Blocked sections were incubated in primary antibody dilutions overnight at 4°C, followed by PBS rinse. Anti-mouse Alexa-Fluor 568 (1:1000, Thermo Fisher Scientific, Australia) and anti-rabbit Alexa-Fluor 488 (1:1000, Thermo Fisher Scientific, Australia) were applied at 37°C for 1 hr, followed by a PBS rinse as secondary conjugated antibodies. Sections were then counterstained with nuclear dye DAPI (1:10,000, Thermo Fisher Scientific, Australia) for 20 min, followed by PBS rinse, coverslipped and imaged using confocal microscopy (TCS SP5, Leica, Germany).

### Image analysis and quantification

Confocal images of stained tissue sections were taken using a Leica SP5 confocal microscope. During imaging, Z-stacks with a step size of 1.5 μm were acquired for each region of interest through the entire tissue section thickness (30 μm), then merged post-process with ImageJ software (National Institutes of Health, US) using maximum intensity projection. In addition to fluorescence images, corresponding bright field images were taken for implanted scaffolds and pseudo-colored in red. Microglial response following scaffold implantation was evaluated in terms of Iba1^+^ pixel intensity profiles from the scaffold/tissue interface at both the tissue and scaffold sides for 30 μm. For each measurement, a 30 μm long line (50 μm line width) was drawn perpendicular to the scaffold/tissue interface. The resulting accumulated pixel intensity per 50 μm was plotted as a histogram of 5 μm intervals. Iba1^+^ average intensities of gP6 at Week 1 and 3 and P6 at Week 3 was compared using 1-way ANOVA (paired data, with Friedman post-test, GraphPad Prism v6.01). GFAP expression level was quantified in terms of GFAP^+^ pixel coverage percentage of a randomly chosen region of interest. Briefly, auto-threshold (based on IsoData algorithm) was used in ImageJ to measure the GFAP^+^ pixel percentage in 30 × 30 μm boxes within the scaffold and adjacent regions. Given the relatively uniform distribution of astrocytes in the tissue adjacent to control and test implants, pixel percentage was measured in 150 μm (from tissue/scaffold boundary) × 50 μm regions to represent a boundary view of inflammatory status in surrounding tissue. The degree of decline in GFAP^+^ intensity was assessed from Week 3 to Week 7 and these values were compared (Mann-Whitney test). In all cases, statistical significance was accepted at P-values <0.05.

## Results and Discussion

### Graphene implants reduce microglial activation/macrophage infiltration

**Fig 1:**
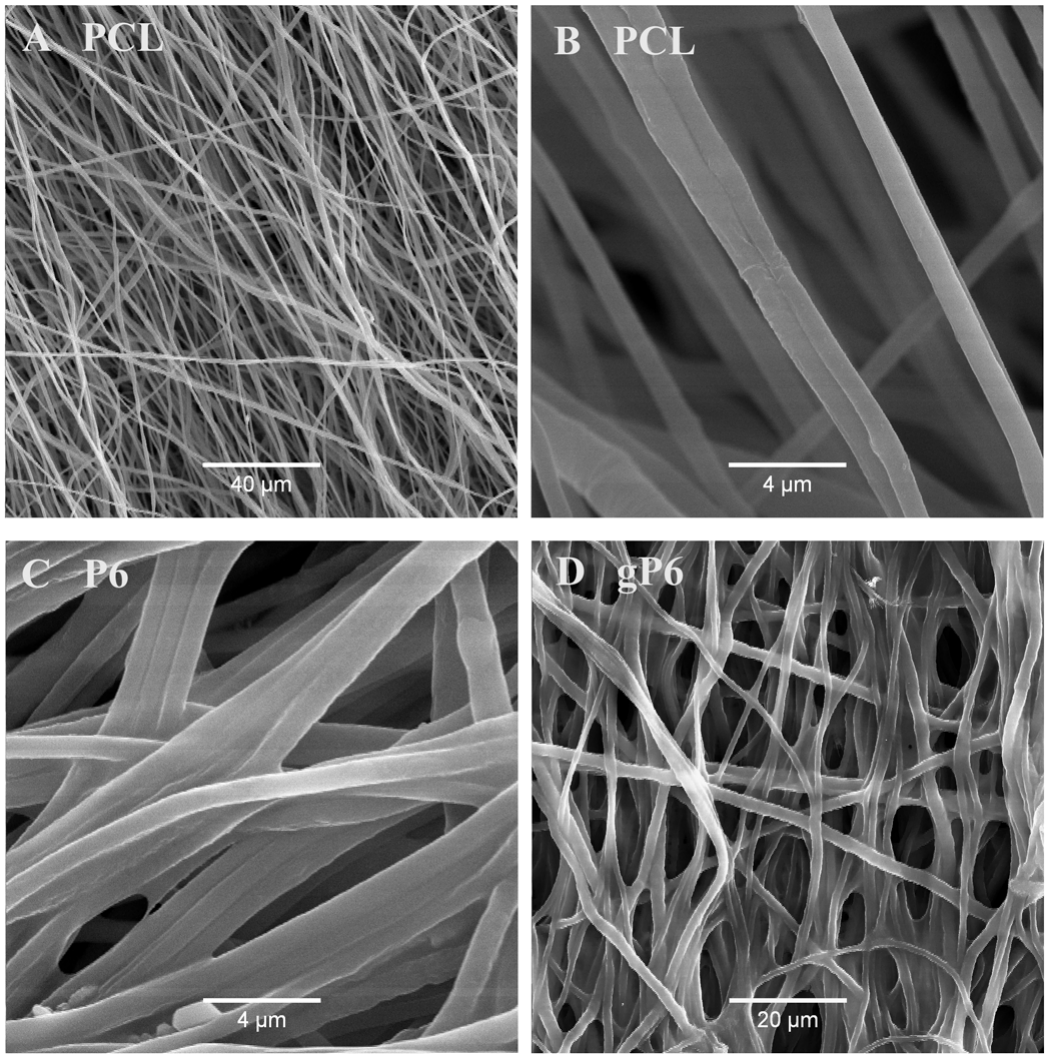
Microstructure of electrospun PCL microfibers and polyelectrolyte modified fibers with and without graphene. SEM images showing the microstructure of (A, B) PCL microfibers, (C) P6 and (D) gP6 modified microfibers. Partially aligned smooth microfiber morphology was revealed in all images at different magnifications.

During the graphene polyelectrolyte growth, electrostatic attraction between positively charged PEI primer on PCL microfibers and negative charged graphene flakes/heparin mixture is the major initial driving force (interactions with microfibers) for the deposition process [37]. The microstructures of electrospun PCL (Fig 1A and 1B) and surface modified P6 and gP6 were reported previously [14,37]. Microfibers (mean diameter 1.1 ± 0.3 μm) exhibited a smooth surface following functionalization with open and interconnected pores after 6 bilayers of LbL / gLbL deposition (Fig 1C and 1D). The single wall thickness of the rolled scaffold implant was 41 ± 8 μm, which was determined from bright field images of the tissue sections with implants.

**Fig 2:**
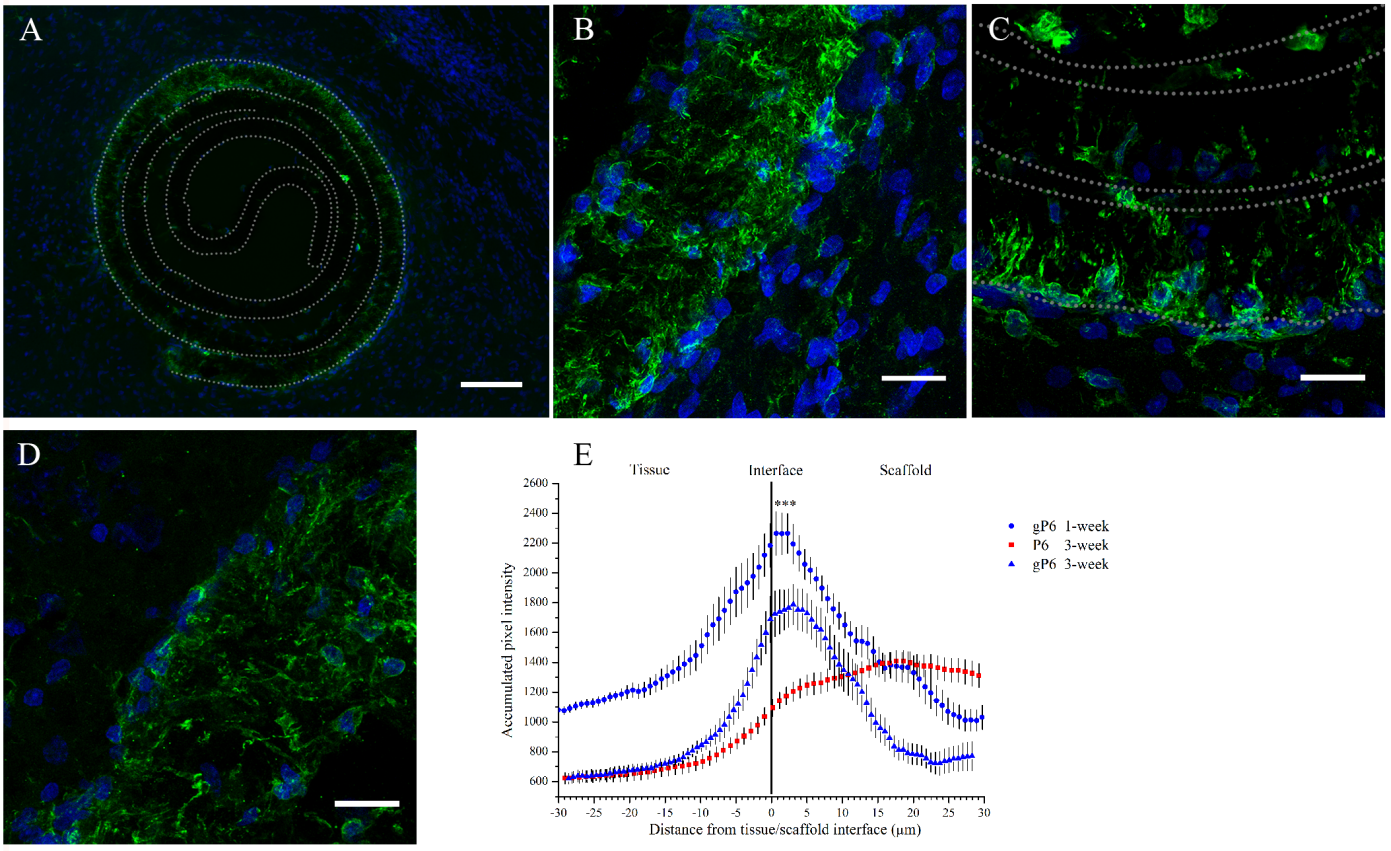
Microglial response to graphene-free or—inclusive PCL scaffold implantation at Week 1 and Week 3. (A) Image shows an overview of microglial infiltration into the outer shell of a gP6 scaffold at Week 3, and gP6 at high magnification at (B) Week 1 and (C) Week 3. (D) Graphene-free P6 scaffold at Week 3 (Green: Iba1^+^ microglia cells, blue: DAPI stained nucleus). (E) Microglial profile across the tissue/scaffold interfaces (*** p<0.001, n = 4). All brain tissue sections were collected on the transverse plane. Scale bar for (A) represents 100 μm; Scale bar for (B, C and D) represents 20 μm. Error bar in (E) shows standard error of the mean.

A typical CNS inflammatory response was characterized histologically by the infiltration of mobile cells of the innate / adaptive immune system [39,40]. Here, the infiltration pattern and protein expression (Iba1+) of activated microglia/peripheral macrophages was employed to reflect the inflammatory progress following implantation. One week following scaffold implantation, Iba1^+^ microglia showed a uniform pattern of distribution along the contour of both gP6 (Fig 2A) and P6 scaffolds (S1 Fig). Microglia had only infiltrated into the outermost layer of both scaffolds, with limited in-growth into the innermost layers of the scrolls (Fig 2B). By Week 3, both the ingrowth depth and process density declined remarkably for the gP6 test implants (Fig 2C). In contrast, microglial processes remained fully penetrating of the outermost scaffold layer in P6 control implants at the same stage (Fig 2D). This time frame of microglia response is well in line with previous reports, identified to occur as early as the first 24 hours, with a peak after 1 week, followed by gradual decline [41]. At Week 1, microglia showed more distinguishable individual morphology in the scaffolds, where activated microglial morphology can be identified. Whole cell migration into graphene containing gP6 test scaffolds was occasionally observed (Fig 2C), while no phagocytic microglia were identified (S2 Fig).

Iba1^+^ intensity profile of gP6 implants at Week 1 and Week 3 had a normally distributed shape (Fig 2E), where the intensity peak occurred at 3 μm into the gP6 scaffolds, and then quickly declined in both scaffold and tissue directions, eventually to a similar level at both sides. At 3 weeks post-surgery, gP6 scaffolds showed significantly lower (p < 0.001, paired ANOVA with Friedman post-test) Iba1 expression compared to the level at Week 1, which is evidenced by a vertical down-shift of Iba1^+^ intensity profile from Week 1 to Week 3 (Fig 2E). Unlike what was observed with the gP6 scaffolds, the microglial response to P6 implants was moderate at the tissue/scaffold interface, but then increased with further penetration into the scaffold, peaking at 20 μm of depth. Moreover, the Iba1^+^ expression in P6 scaffolds did not decline to the same level as the tissue side; instead, it remained relatively high throughout the entire scaffold layer. Despite the differences in Iba1^+^ intensity profile at Week 3 between P6 and gP6 implants, no significant difference in Iba1^+^ intensity was observed. Since microglial morphology is closely related to biological function [42], it is clear that in both P6 and gP6 implants, microglia were in an active state, as revealed by a less ramified but thicker processes than in the resting stage, without the enlarged appearance of phagocytic cells. The results indicate a possible persistence of a mild inflammatory stage by Week 3 in P6 and gP6 implants. Although the pro- or anti-inflammatory phenotype of the activated microglia in the scaffolds could not be determined, no evidence was found at Week 3 in these implants that activated microglia cells triggered the cascade of secondary tissue damage and glial scarring, which is commonly observed during this stage of brain injury [43]. The significant decrease in microglial ingrowth and density in gP6 implants from Week 1 to Week 3 suggests that the graphene caused a reduction in microglial activation/macrophage infiltration, constraining them near the tissue/scaffold interface at a later stage of inflammation. This could possibly be due to the lower pro-inflammatory cytokine or reactive oxide species being secreted, which is attributed to the three dimensional presentation of graphene on microfiber surfaces [44]. This could subsequently reduce the self-propelling cycle of microglia activation that shortens the chronic inflammation period.

### Astrocyte morphology and infiltration patterns

**Fig 3:**
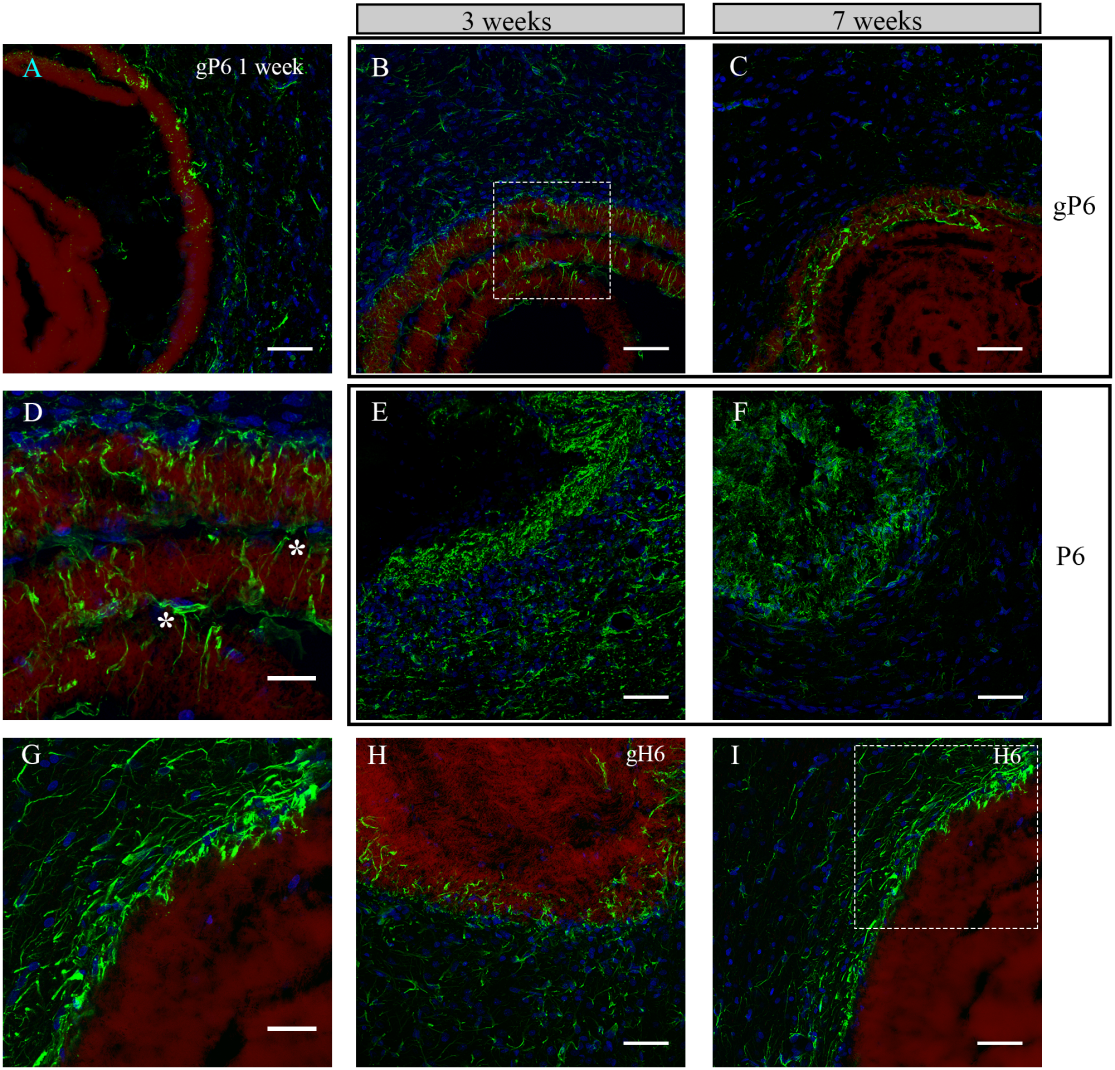
Astrocyte morphology and infiltration at different time points following H6, P6, gP6, gH6 scaffolds implantation. Astrocyte/gP6 scaffolds interaction at Week (A) 1, (B) 3 and (C) 7; Astrocytes/P6 scaffolds interaction at Week (E) 3 and (F) 7; Astrocyte process infiltration into (H) gH6 at Week 3 and (I) H6 at Week 7. (D, G) detailed astrocyte morphology of the dash-box indicated area in (B, I) respectively. Green: GFAP positive astrocytes, blue: DAPI stained nucleus, red: surface functionalized scaffolds. * indicates astrocytes that bridge a gap between two scaffold layers in (D). All brain tissue sections were collected on the transverse plane. Scale bar for (D, G) represents 20 μm; for all other images represents 50 μm.

Ingrowth patterns of astrocytes were examined in gP6, P6, gH6 and H6 implants at Week 1, 3 and 7 post-surgery. At Week 1, a few astrocytes could be observed, scattered in both tissue and scaffold phases, showing minimum infiltration into the gP6 scaffold (Fig 3A). No GFAP^+^ cells were observed to have aggregated at the tissue/scaffold interface. These observations suggest that the implanted scaffolds did not trigger a significant immune response at an earlier time point, nor increase the level of activation, in light of the typical activation onset time for astrocytes of approximately 2 weeks after implantation [45]. At Week 3, astrocyte process infiltration was markedly increased into multiple layers of the gP6 scaffold (Fig 3B). Interestingly, both GFAP^+^ cell bodies and their processes (Fig 3D), highlighted by left and right asterisk respectively) were found within the inter-layer gap of the scaffold. Astrocytes in the adjacent tissue of the gP6 scaffold again showed no evidence of accumulation at the scaffold/tissue interface throughout Week 1 to 7. By Week 7, although GFAP^+^ processes remained in the outer layers of the gP6 scaffolds, the number of astrocytes in the adjacent tissue dropped significantly (Fig 3C) and no glial scarring was observed at the tissue/scaffold interface.

In contrast to the astrocyte response to gP6 implants, GFAP^+^ processes/cells in either P6 implanted scaffolds or adjacent tissue revealed remarkably higher density (Fig 3E and 3F), such that individual astrocyte morphology could no longer be distinguished, possibly reflecting the increased exposure to the cell-adhesive properties of PLL, as described previously [14]. From Week 3 to Week 7, there was a decline in the number of astrocytes in both scaffold and striatum, which was more pronounced on the tissue side (Fig 3F), where lightly ramified astrocytes were observed. For heparinized scaffolds, astrocyte density was highest near the outermost layers of the gH6 and H6 scaffolds at Week 3 and 7 respectively (Fig 3H and 3I). However, infiltration was only observed in the gH6, where GFAP^+^ processes had inserted randomly along the contour of gH6 implants (Fig 3G). There was no obvious infiltration in the H6 scaffolds (Fig 3I), with process alignment parallel to the implant and aggregation in the adjacent tissue resembling a glial scar. It thus appears that astrocyte infiltration and morphology switch completely from an ingrowth pattern to a glial scar pattern, depending on whether the terminating layer contained adhesive PLL (P6) or non-adhesive heparin (H6) [46]. The presence of graphene improved in-growth in the latter case, despite the same scaffold microstructure in both. In addition, GFAP immunostaining of the SVZ gP6 implants at Week 3 revealed similar morphology and infiltration as the striatum gP6 implants (S3 Fig). In both implant locations, no SMI-32^+^ cells were identified within the gP6 scaffolds.

### Astroglial protein expression variability

**Fig 4:**
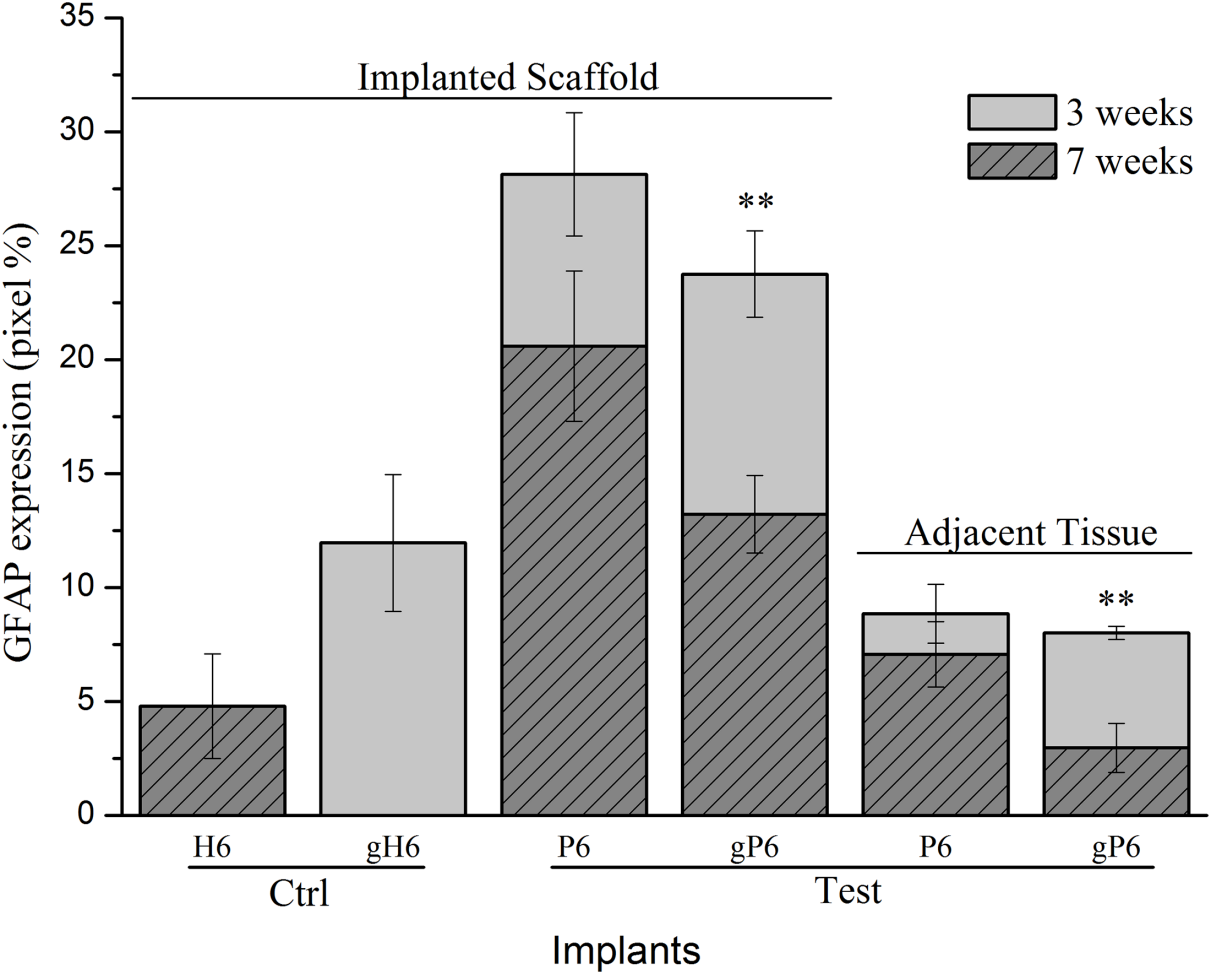
GFAP expression at different time points in the outer layer of scaffolds and in adjacent tissue. (150 μm from tissue/scaffold boundary) in terms of GFAP^+^ astrocytes occupied pixel percentage (pixel%). * (p<0.05, n = 4) indicate significant difference in GFAP expression between Week 3 and 7, within scaffold or tissue for gP6 implants. Error bar shows standard error of the mean.

GFAP protein levels at Week 3 and 7 were detectable in both the outermost layer of implanted scaffolds and the adjacent striatum tissue (Fig 4). Both P6 and gP6 scaffold implants demonstrated lower GFAP expression in the adjacent tissue, compared to within the implanted scaffold at Week 7 (7.1% and 3.0% for adjacent tissue; 20.6% and 13.2% for within the scaffold, respectively). gP6 scaffolds also showed lower levels of GFAP expression compared to their respective P6 controls, at both Week 3 (23.8% vs. 28.1%) and Week 7 (13.2% vs. 20.6%) within the implanted scaffolds. Similarly, gP6 scaffolds showed lower levels of GFAP at both week 3 (8.0% vs. 8.9%) and week 7 (3.0% vs. 7.1%) in the adjacent striatal tissue. Overall, average GFAP expression levels were 1.6 and 2.4 times lower in the gP6 implant and adjacent tissue respectively, compared to P6 control groups. These results suggest that the incorporation of graphene into the polyelectrolyte multilayer coating renders implants more biocompatible. Notably, reduction in GFAP level is not apparently due to inhibition of astrocyte infiltration, but rather the relatively low density of reactive astrocytes. Graphene incorporation changes the tissue-scaffold interaction dramatically to a pattern with near doubling of GFAP intensity for gH6 implants compared to H6 scaffolds, though with moderate infiltration into the outermost scaffold layer, and no scaring in the adjacent tissue.

Considering the time course of astrocyte activation, GFAP expression level declined from Week 3 to Week 7 within both gP6 and P6 implanted scaffolds and in the adjacent tissue. There was a significant decrease in GFAP protein expression (p < 0.01, Mann-Whitney test) with gP6 scaffolds from Week 3 to Week 7 in both the scaffold material and adjacent tissue (23.8% to 13.2% in the scaffold; 8.0% to 3.0% in the adjacent tissue), however the decline from Week 3 to Week 7 in P6 controls was not statistically significant. Both gH6 and H6 heparinized groups showed much lower GFAP intensity compared to gP6 or P6 poly-lysinized groups (12.0% for gH6 (Week 3) vs. 4.8% for H6 (Week 7)) in the scaffold materials, while the adjacent tissue levels were not compared, due to difficulty in quantitating glial scarring and non-uniform distribution of astrocytes in the gH6 and H6 groups. The higher GFAP levels in gH6 scaffolds compared to H6, which directly reflects the increased astrocyte infiltration, may be influenced by the charge coupling or local ion trapping properties of graphene, which may impact on astrocyte processes via mechanisms such as calcium dependent signaling [47]. Alternatively, graphene hydrophobicity, in combination with variable PEM surface charge, may modulate short range attraction forces (e.g. H-bonding etc.) [48], to preferentially adsorb proteins like pro-inflammatory cytokines, depending on the nature of their hydrophobicity and charge [49]. Potential conformational changes of absorbed proteins due to strong hydrophobic interaction between hydrophobic domains of the proteins and underlying graphene [34,48,50,51] may also change local stimulation cues, resulting in altered guidance cues.

### Control of directional guidance of infiltrated astrocytes

**Fig 5:**
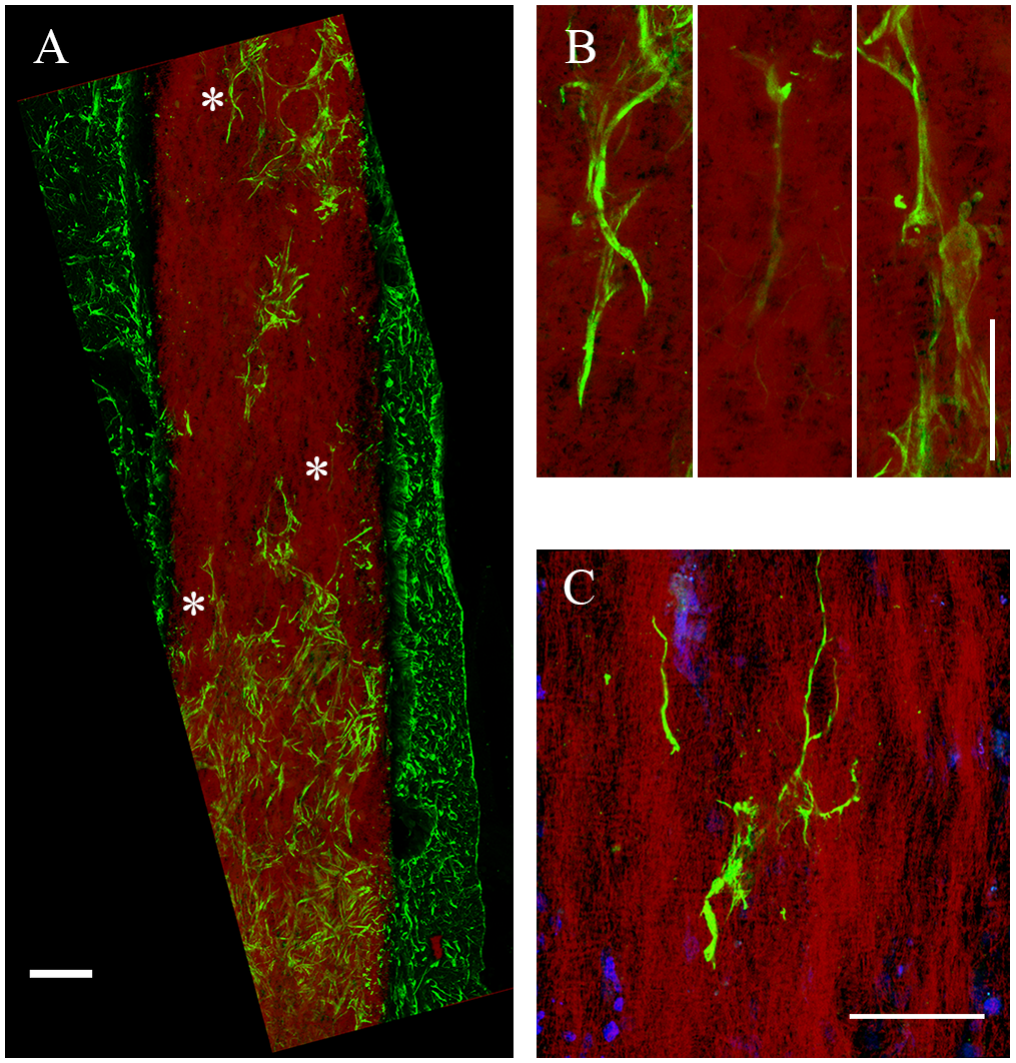
Astrocyte directional growth with a gP6 scaffold following process infiltration. (A) astrocytes and processes change their cell/process alignment to the microfiber direction to follow fiber alignment after infiltration into gP6 implant. (B) Enlarged areas in (A, marked by *) indicating astrocytic processes alignment along the vertical axis. (C) Multiple process alignment of a single astrocyte within the scaffold. Brain tissue sections were collected in the sagittal plane. Scale bars in (A) and (B, C) represent 100 and 20 μm respectively.

The directionality of astrocyte infiltration was not limited radially in the transverse plane. We also observed astrocyte alignment with the long axis of gP6 implants, either parallel (Fig 5A and 5B) or partially parallel (Fig 5A bottom) to the fiber direction. This process alignment was more pronounced for astrocytes found deep in the scaffold interior. At or near the tissue/scaffold interface, little alignment of penetrating processes was observed. In addition, regions of the brain with lower astrocyte density (Fig 5A top) revealed higher alignment with the long axis of the scaffold, compared with regions with higher cell density (Fig 5A bottom). Individual whole cells were also observed with processes extending along the direction of single microfibers (Fig 5B). Thus, GFAP^+^ cell process alignment with scaffold microfibers appears to depend on the relative number of cell-cell and cell-scaffold interactions. The latter is significant in relation to the effect of contact guidance cues on astrocyte migration, especially in regions where cell-cell interactions are less dominant, and confirms the strong radial ingrowth observed above as well as in previous reports [52,53].

### Neuroblast migration is supported by graphene scaffolds

**Fig 6:**
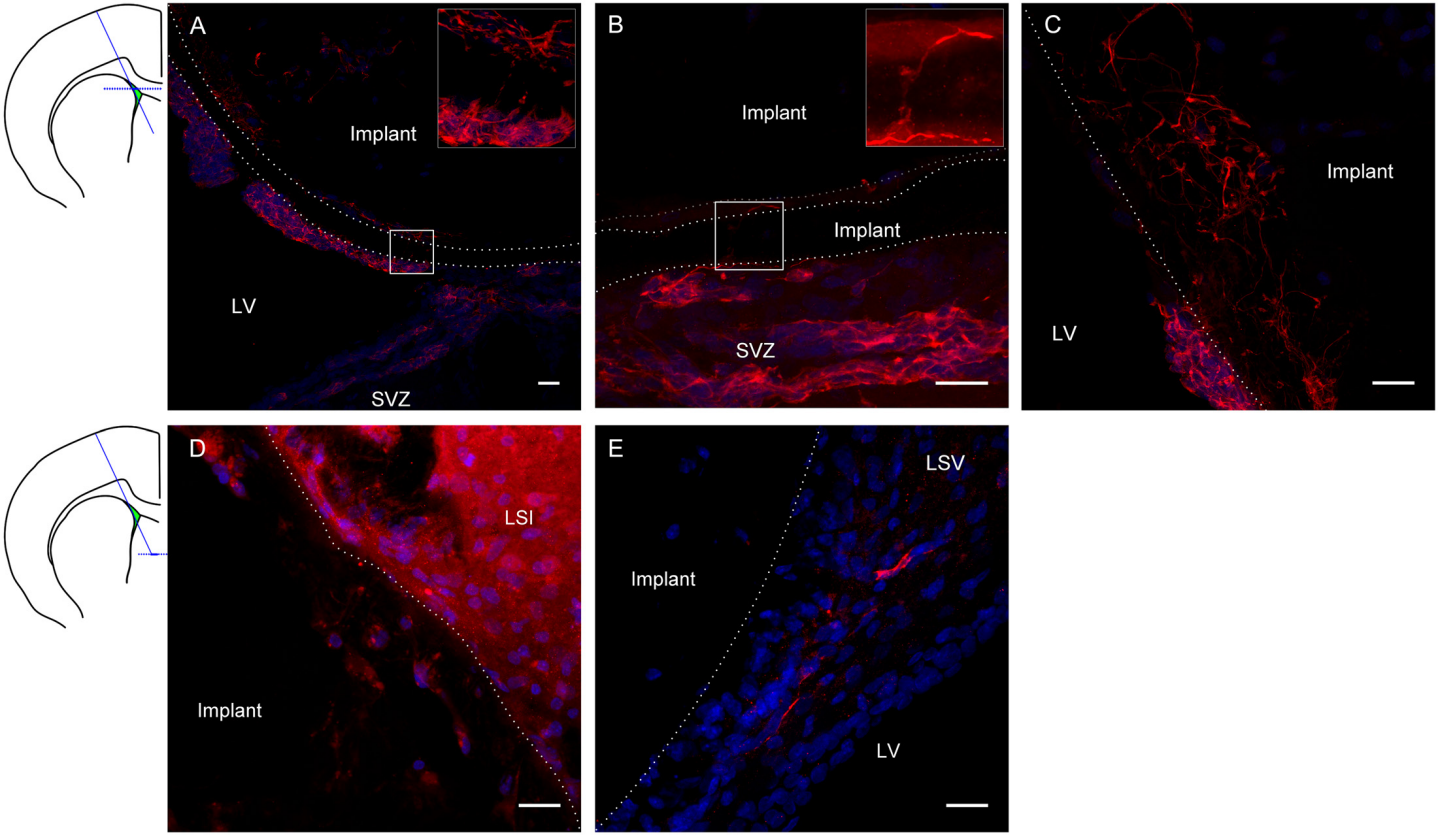
Neuroblast migration/integration along/with gP6 implants at Week 3. Immunostaining images show DCX^+^ neuroblast (red) and nuclei (DAPI, blue) of tissue sections at the transverse plane. Schematic coronal brain section indicates the gP6 implantation track and locations of A-C, D-E tissue sections respectively. gP6 implant boundary is marked by dotted line. (A) gP6 implant in LV, bottom right part of the outermost scaffold layer is in direct contact with SVZ. Insert enlarges the box region showing neuroblast process across the entire scaffold layer. (B) Neuroblasts migrate along the scaffold surface/inter-layer gap and (C) processes infiltrating into the scaffold layers. Neuroblasts are identified in deeper sections of the gP6 implants either (D) within the scaffold or (E) close to implant in the LSV. LV: lateral ventricle, LSI: lateral septal nucleus, intermediate part, LSV: lateral septal nucleus, ventral part. Scale bars represent 20 μm for all images.

Based on the apparently lower inflammatory response, gP6 scaffolds were chosen as the optimal candidate to investigate whether they could act as conduits to direct neuroblast migration from the SVZ, along the scaffold, to other potential target regions of the adult brain. When the gP6 scaffold was implanted to intercept the SVZ (Fig 6A–6C), DCX^+^ neuroblasts migrated along the scaffold surface, 30 degrees to the natural trajectory of the neuroblasts. These data are similar to our previous observation with PCL scaffolds releasing a small molecule BDNF-mimetic [7]. The dotted line shows the outermost layer of the gP6 scaffold in Fig 6A, where a majority of the implant settled in the lateral ventricle, with the bottom right segment in direct contact with SVZ. By Week 3, most DCX^+^ cells were located in three different regions, including (i) the scaffold inter-sheet cavity (Fig 6A and 6B); (ii) the lateral ventricle, adherent to the implant, and (iii) the gP6 scaffold outer layer. In the first instance, when neuroblasts had migrated to the interlayer cavity and were in close contact with the scaffold, DCX^+^ processes mainly extended parallel with the scaffold layer contour (Fig 6A insert, Fig 6B), even though the scaffold microfiber alignment direction was perpendicular to the tissue section plane. Occasionally, some DCX^+^ processes were able to find the shortest path to infiltrate through intervening gP6 scaffold layers (Fig 6A insert, Fig 5B insert). This observation suggests that gP6 supports local interactions with neuroblast growth cones. The underlying mechanism for this is not clear, however, this observation is similar to the migration pattern along blood vessels of the rostral migratory stream [54]. Interestingly, neuroblast cells also migrated along the outer surface of gP6 scaffolds toward the lateral ventricle (Fig 6A bottom-left segment of implant). However, neuroblast processes were more randomly orientated, compared to those in between scaffold layers, which could be due to stronger cell/scaffold interactions and interlayer space restrictions. DCX^+^ cells that had infiltrated into the gP6 layers displayed random morphology, with long processes. Considering tissue sections in ventral regions (Fig 6D and 6E), gP6 scaffolds were localized to the LSI and LSV regions of the rat brain, which is some distance from the direct source of neuroblasts. A small number of DCX^+^ cells with short process were also found within the implant (Fig 6D), although these revealed much lower fluorescent intensity, possibly indicating finer processes. This observation demonstrates the migration of neuroblasts along the implant from dorsal segments ventrally (as shown in Fig 6A–6C, about 1.2 mm). In contrast to the present study, we showed previously that DCX^+^ cells that migrated into BDNF-loaded PCL scaffolds were no longer visible by Week 3 [7]. This may suggest that the incorporation of graphene is able to maintain the undifferentiated stage of neuroblast for a longer period. The gP6 implants also attracted neuroblasts to the near surface of scaffolds (Fig 6E), possibly branching from SVZ region from a long distance via injury initiated migration mechanisms [55].

## Conclusion

Graphene-LbL surface modification of PCL scaffolds suppresses microglia and astrocytes activation and the number of infiltrated macrophages after implantation. Microglia/macrophage were markedly lower in gP6 groups by Week 3 compared to P6 implants, of which no glial scarring was observed in the surrounding tissue. Different surface functionalization chemistries had no effect on the onset time of astrocyte activation. However, gP6 scaffolds notably lowered the number of activated astrocytes in both tissue and implants from Week 3 to Week 7. On the other hand, graphene encouraged astrocyte infiltration into gP6 implants. This enhanced infiltration was also observed in gH6 implants, which exposed non-adhesive heparin surface molecules. Guidance cues of the aligned gP6 scaffold had an effect on the directional growth of infiltrated astrocytes. gP6 implants successfully promote the growth of neuroblasts from SVZ, with directional guidance achieved through the scaffold. Further studies will be required to confirm the differentiation status of these migratory neuroblasts, as well as their capacity to traverse variable distances, and to what extent inflammatory cells may mediate this process.

## Supporting Information

S1 Fig Microglial infiltration into P6 implant at Week 3. Microglia infiltrated into P6 implant outermost layer along implant contour. Limited ingrowth to the inner layers was observed. Scale bar represents 50 μm.
(TIFF)

S2 Fig Microglia whole cell migration into gP6 implant at Week 1. Whole cell migration of microglia into gP6 scaffolds is highlighted in green. Microglia in the scaffold showed multiple processes, similar to ramified morphology. Scale bar represents 20 μm.
(TIFF)

S3 Fig Astrocytes infiltration into gP6 scaffolds implanted in the SVZ at Week 3. Astrocytes infiltrate into the outermost layer of gP6 implant. cc: corpus callosum. Scale bar represents 20 μm.
(TIFF)
