# Supplementary figures and images for: Graphene Functionalized Scaffolds Reduce the Inflammatory Response and Supports Endogenous Neuroblast Migration when Implanted in the Adult Brain
Source: PLoS One. 2016 Mar 15;11(3):e0151589. doi: 10.1371/journal.pone.0151589 (PMC4792446; doi:10.1371/journal.pone.0151589)

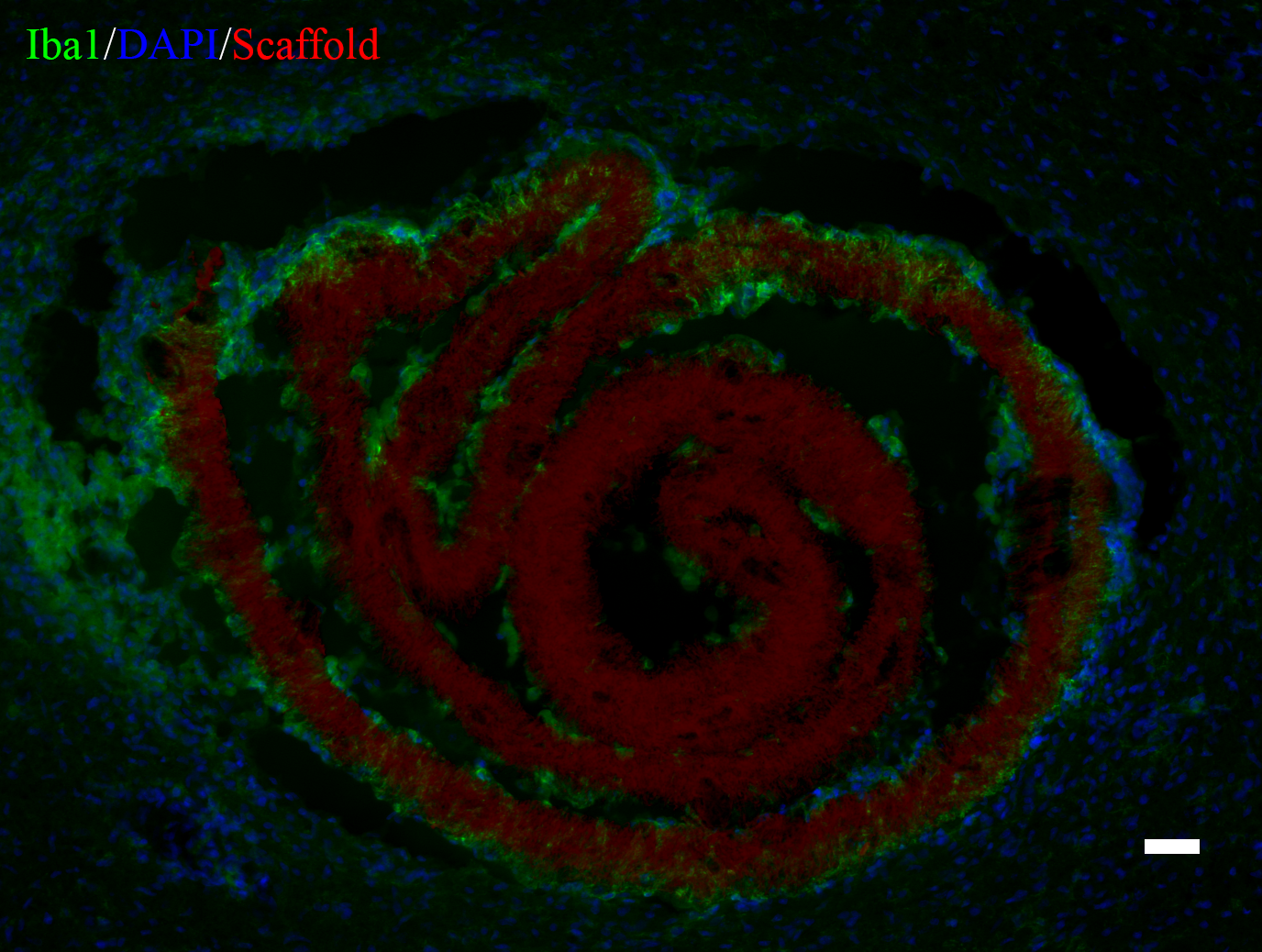

Supplement: S1 Fig — Microglia infiltrated into P6 implant outermost layer along implant contour. Limited ingrowth to the inner layers was observed. Scale bar represents 50 μm. (TIFF) [file pone.0151589.s001.tiff]

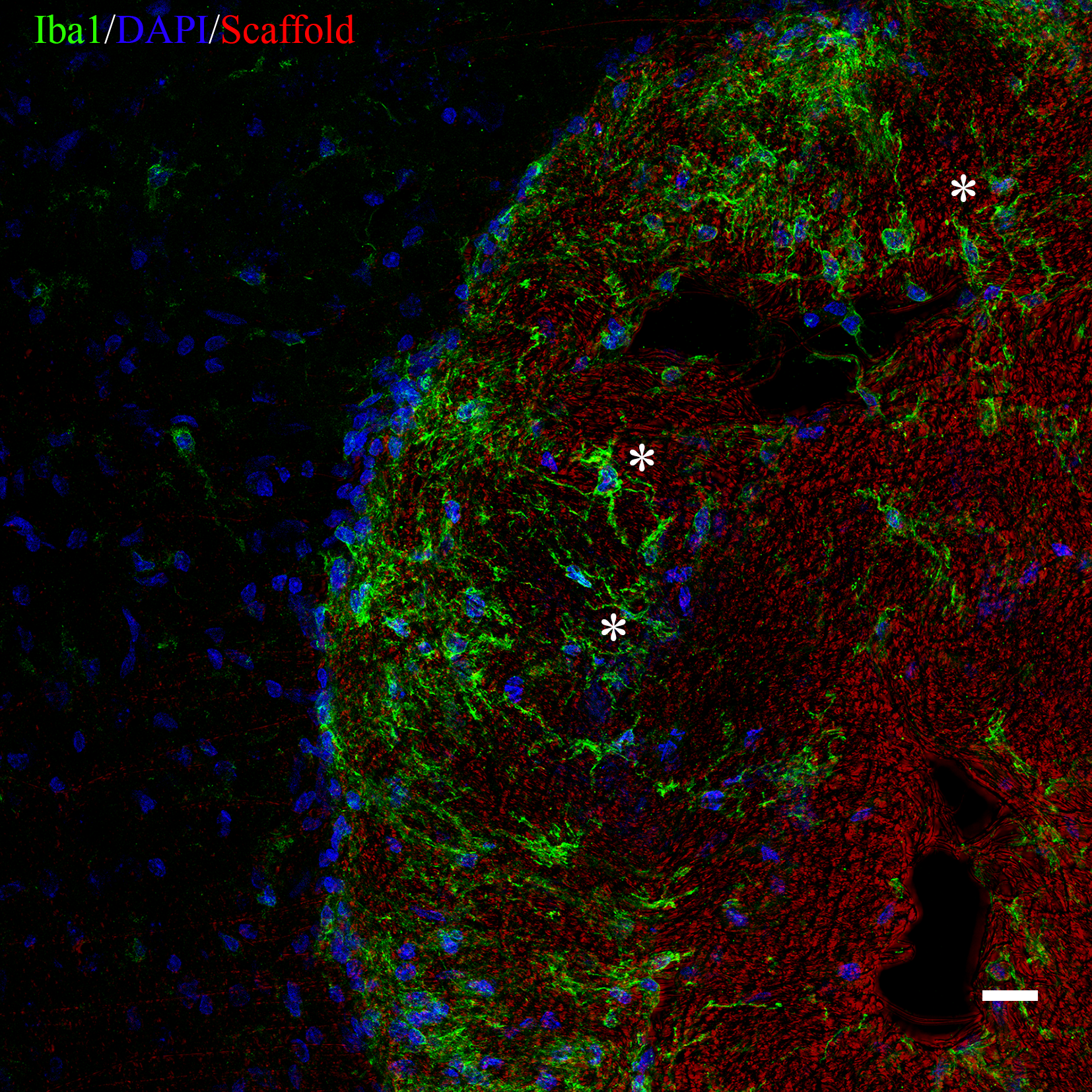

Supplement: S2 Fig — Whole cell migration of microglia into gP6 scaffolds is highlighted in green. Microglia in the scaffold showed multiple processes, similar to ramified morphology. Scale bar represents 20 μm. (TIFF) [file pone.0151589.s002.tiff]

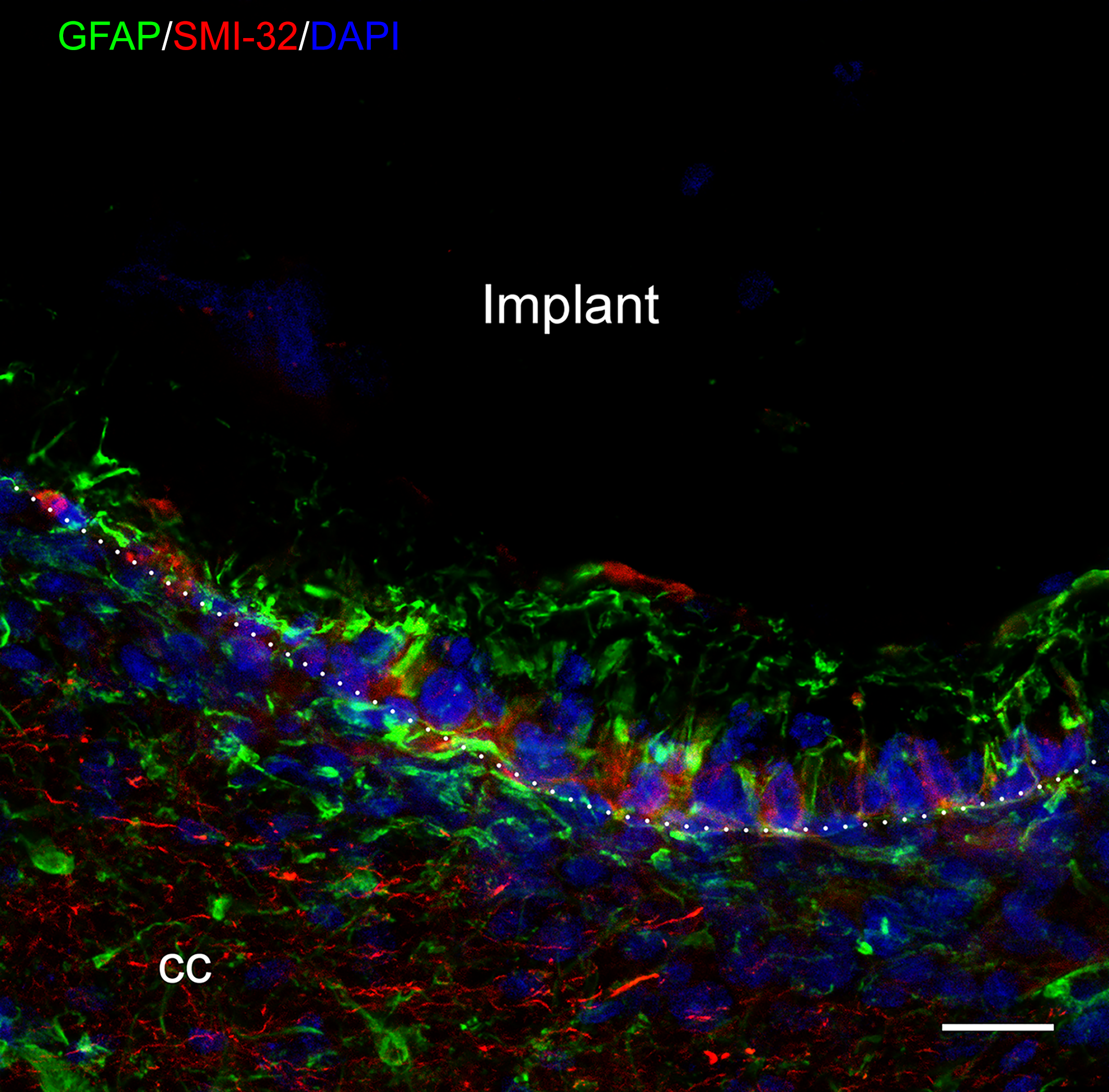

Supplement: S3 Fig — Astrocytes infiltrate into the outermost layer of gP6 implant. cc: corpus callosum. Scale bar represents 20 μm. (TIFF) [file pone.0151589.s003.tiff]
